# Roles and Mechanisms of TRIM Family Proteins in Inflammation in the Brain and Beyond

**DOI:** 10.3390/ijms27031135

**Published:** 2026-01-23

**Authors:** Tatiana Gerasimova, Alisa Kotok, Sofia Saltykova, Ekaterina Stepanenko, Artem Eremeev, Ekaterina Novosadova, Vyacheslav Tarantul, Valentina Nenasheva

**Affiliations:** 1 Laboratory of Translational Biomedicine, Lopukhin Federal Research and Clinical Center of Physical—Chemical Medicine of Federal Medical Biological Agency, 119435 Moscow, Russia; ger.tp@mail.ru (T.G.); katishsha@mail.ru (E.S.); art-eremeev@yandex.ru (A.E.); novek-img@mail.ru (E.N.); valnenasheva55@gmail.com (V.N.); 2 Laboratory of Molecular Neurogenetics and Innate Immunity, National Research Center “Kurchatov Institute”, 123182 Moscow, Russia; sssaltykova1999@gmail.com (S.S.); ninaslava130@yandex.ru (V.T.); 3 Center for Genetic Reprogramming and Gene Therapy, Lopukhin Federal Research and Clinical Center of Physical—Chemical Medicine of Federal Medical Biological Agency, 119435 Moscow, Russia

**Keywords:** TRIM proteins, inflammatory signaling, neuroinflammation, peripheral organ inflammation

## Abstract

Neurodegeneration is closely linked to neuroinflammation and is frequently accompanied by comorbidities with inflammatory features. Tripartite motif (TRIM) proteins are known to play an important role in innate immunity and inflammatory signaling in various tissues and organs of the body, including the central nervous system. Among the main cell types of the brain, TRIMs’ functions in microglia are largely associated with the regulation of intracellular inflammatory signaling, while in neurons they mainly relate to cell survival and oxidative stress. Data concerning TRIMs’ activity in astrocytes remain limited. Many TRIM proteins exert similar pro- or anti-inflammatory effects in neuroinflammation and in other inflammatory disorders in the body, although for some members their roles are reported to be opposite, contradictory, or insufficiently characterized, highlighting the need for further research. The aim of this review was to summarize published data on the common mechanisms of TRIMs’ actions as modulators of inflammation, and compare available reports in the context of neuroinflammation and peripheral inflammatory pathologies. We suggested that such an analysis may be valuable for guiding future research—both by identifying existing gaps in knowledge and by supporting the rational selection of specific TRIM proteins for investigation as therapeutic targets, with careful consideration of their systemic effects.

## 1. Introduction

The nervous and immune systems of the organism actively cooperate and affect each other. The dysregulation of such communication may lead to severe central nervous system (CNS) impairment [1]. The term “neuroinflammation” refers to inflammatory processes in the brain tissue aimed both at maintaining CNS homeostasis or respond to injury and disease. While acute neuroinflammatory responses may be protective, facilitating the clearance of pathogens and debris, chronic or excessive inflammation can lead to neuronal dysfunction and degeneration. Neuroinflammation occurs not only in traumatic and infectious brain pathology, but also in a wide range of neurodegenerative disorders (i.e., Alzheimer’s disease (AD), Parkinson’s disease (PD), amyotrophic lateral sclerosis), and is currently seen as one of the mechanisms behind their onset [2]. Neurodegenerative diseases are frequently accompanied by comorbidities that are also characterized by a significant inflammatory component, including type 2 diabetes, obesity, atherosclerosis, etc. [3]. These pathologies often share common dysregulated inflammatory pathways with neurodegeneration.

The tripartite motif (TRIM) protein family consists of 77 members that primarily function as RING-type E3 ubiquitin ligases. They contain the tripartite motif, a protein structure that comprises a RING-finger domain, a B1-box and/or a B2-box domain, and a coiled-coil region. TRIM proteins take part in various important biological processes, such as immunity, apoptosis, and intracellular signaling. TRIMs are actively involved in the regulation of the innate immunity in antiviral response [4] and other different pathological states [5], modulating pattern recognition receptors, adaptor proteins, kinases, and transcription factors [6]. At the same time, they are engaged in the functioning of the nervous system, including its development [7,8]. Neuroinflammation serves as a vital link between the nervous and immune systems, making it a promising area of research.

In this regard, the present review aims to observe the known data on TRIM proteins’ participation in inflammatory signaling in general, and to identify correlations between the existing evidence on their role in neuroinflammation and inflammation in other organs and tissues in the body. The literature search was carried out in three databases (PubMed, Scopus, and Web of Science) in peer-reviewed sources, tracing relevant studies up to and including 2025. The primary search was focused on TRIM proteins that are linked to neuroinflammation, using key words such as “TRIM proteins”, “neuroinflammation”, “neurodegenerative diseases”, etc. As a result, a list of neuroinflammation-related TRIMs was formed. Then, a literature search was conducted for each of the members of the list, focusing on their roles in other types of inflammation. The selected TRIM proteins were afterwards divided into five groups (TRIMs with proinflammatory properties, TRIMs with anti-inflammatory properties, TRIMs with opposite effects in the CNS and in other organs and tissues, TRIMs with divergent evidence, TRIMs with insufficient data) based on their action in neuroinflammation versus other types of inflammation.

## 2. TRIM Protein Structure and Function in Inflammation

The common TRIM structure consists of the RING domain responsible for the E3 ubiquitin ligase activity, B-box domains engaged in higher complex formation, and a coiled-coil region that is involved in multimerization. The current literature abounds with evidence of particular targets associated with inflammation that are ubiquitinated by different TRIM family members, namely, TAK1 (TRIM8) [9], TAB2 (TRIM45) [10], TNF receptor-associated factor 2 (TRAF2) and NF-κB essential modulator (NEMO) (TRIM47) [11,12], and NLRP3 components (TRIM31 [13], TRIM50 [14], TRIM65 [15]). Some TRIM proteins (TRIM14, TRIM16, TRIM20), however, have no RING domain and, thus, do not possess E3 ligase activity [16], and their function in inflammation is associated with other structural regions of the protein.

The 54–136 amino acid N-terminal sequence of TRIM9, which is in between RING and B-box domain [UniProtKB A0A804HIL7_HUMAN], is essential for its suppression of NF-κB signaling [17].

B-box domains are essential for substrate engagement and facilitate protein–protein interactions within the TRIM family. TRIM5α B-box/B-box interactions lead to the formation of a hexagonal net, which is crucial for recognizing HIV-1 capsids [18]. B-boxes of TRIM1 and TRIM18 have been suggested to act as target recognition motifs [19].

The coiled-coil (CC) domain is responsible for oligomerization. Dimers are often needed for basic activity, and higher oligomers for pathogen recognition. The avidity of TRIM5α CC dimers is sufficient to allow the detection of virus capsid binding in vitro [20]. A minimum of two TRIM25 CC-dimers is required to generate a catalytically active E3 ligase [21]. TRIM50 induces NLRP3 oligomerization via its CC domain [14]. Both B-box and CC domains contribute to the TRIM62-driven activation of transcription factors nuclear factor-kappa B (NF-κB) and activating protein-1 (AP-1) [22].

Based on different C-terminal structures, TRIM proteins are further divided into 11 subfamilies. The most common one is the PRY-SPRY domain (also known as the B30.2 domain), covering more than half of known TRIMs. This domain is required for AP-1 activation by TRIM15 [22] and mediates the inhibition of inflammasome activity by TRIM20 through interactions with NLRP3, caspase-1, and pro-IL-1β [23]. TRIM16 uses its SPRY subdomain to engage with p62 and NRF2 [24]. Two binding pockets—one in the PRY and the other in the SPRY subdomains—enable TRIM21 to bind to IgG heavy chains [25]. The PRY-SPRY domain of TRIM21 interacts with DDX41, prompting K48-linked polyubiquitination and the degradation of DDX41, thereby dampening the intracellular DNA-sensing response [26]. The SPRY subdomain of TRIM25 is crucial for the binding and polyubiquitination of RIG-I [21]. TRIM38 mediates the lysosomal degradation of TAB2/3 through its C-terminal PRY-SPRY domain, independently of the RING domain [27]. The C-terminal sequence of TRIM56, but not the RING domain, controls the ability of the protein to promote TLR3 signaling [28].

Other variable C-terminal domains (COS, FN3, ACID, ARF, filamin, NHL, plant homeodomain (PHD), the bromodomain, and the transmembrane region (TM)) are also responsible for the precise diverse substrate specificities of protein–protein interactions [7]. Additionally, they take part in nucleic acid binding [7] and may also determine cellular localization [29].

The joint activity of TRIM domains in different combinations defines the variety of mechanisms that TRIMs perform during inflammation.

## 3. Subcellular Localization of TRIM Proteins Involved in Inflammation

The subcellular localization of TRIM proteins largely determines their functions in the inflammatory response, either as positive regulators of inflammatory signaling or as repressors of pro-inflammatory factors’ expression. For instance, TRIMs localized in the cytoplasm mainly mediate the activation of signaling cascades such as NF-κB and interleukins, and regulate inflammasome assembly [11,30,31]. Nuclear TRIM proteins, by contrast, suppress the transcription of pro-inflammatory genes and directly influence epigenetic regulation [32,33,34]. Mitochondrial and membrane-associated TRIMs perform more specialized functions; those in mitochondria are involved in antiviral immunity (e.g., via MAVS interaction) [35], while membrane-associated TRIM proteins participate in membrane repair and regulate lysosomal degradation [27,36,37]. Some TRIM family members are found in different cellular compartments, carrying out distinct roles depending on their localization. Of particular interest are TRIM proteins capable of dynamic re-localization—they adapt their function by moving between the nucleus, cytoplasm, membranes, and mitochondria in response to stimuli such as cytokines or cellular stress [27,38,39]. The intracellular localization of a protein is closely linked to its specific mechanism of action, as it defines the accessibility of the molecules with which the protein interacts. Thus, spatial and temporal control allows TRIM proteins to finely tune the intensity and duration of the inflammatory response. The Table 1 illustrates examples linking the subcellular localization of TRIM proteins to their biological effects in inflammation.

## 4. Key Molecular Mechanisms of TRIM Proteins’ Action in Inflammation

Most TRIM proteins act as E3 ubiquitin ligases, utilizing their RING domains to mediate the ubiquitination of target proteins important in immune response. Monoubiquitination can influence protein localization and DNA repair, while different types of polyubiquitin chains cause specific cellular outcomes. K48-linked chains typically signal for proteasomal degradation, such as the ubiquitination of IRF3 by TRIM21 [48] and TRIM 26 [49], whereas K63-linked chains promote signaling events, such as the activation of MAVS by TRIM31 [50] and STING by TRIM56 [51]. However, ubiquitination is not the only mechanism of TRIM proteins’ effect. Some of them operate through non-enzymatic means, including direct protein–protein interactions. They influence TLR signaling—both MyD88- and TRIF-dependent branches—by modulating adaptor proteins, kinases, and transcription factors such as NF-κB, AP-1, and IRFs. Certain TRIMs modulate the NLRP3 inflammasome pathway, activating caspase-1 and the release of IL-1β and IL-18. TRIMs are also involved in RIG-I-like receptor and cytosolic DNA sensing pathways, impacting antiviral immunity and the production of inflammatory cytokines. These diverse activities enable TRIM proteins to regulate several key inflammatory signaling pathways.

### 4.1. NF-κB Signaling

The NF-κB pathway is central to inflammation, and regulates the production of pro-inflammatory cytokines. In resting cells, NF-κB p65/p50 is kept in the cytoplasm by its inhibitor, IκBα, and is activated in response to pro-inflammatory stimuli, such as TNF-α, IL-1β, lipopolysaccharide (LPS), and danger-associated molecular patterns (DAMPs), through receptors like TNFR, TLRs, and RAGE. This ligand–receptor interaction triggers a signaling cascade. The IκB kinase (IKK) complex phosphorylates IκBα, marking it for K48-linked ubiquitination and proteasomal degradation by β-transducin repeat-containing protein (β-TrCP). NF-κB p65/p50 subunits translocate into the nucleus, which activates the expression of inflammatory genes and thereby increases the release of inflammatory cytokines. In PD, the number of dopaminergic neurons in the substantia nigra-expressed nuclear p65 was found to be 70 times higher than in age-matched controls [52]. Persistent NF-κB activation in microglia and neurons exacerbates neuroinflammation both in neurodegeneration and acute brain injury [53]. TRIM proteins modulate this pathway at multiple points, including the stabilization of IκBα (TRIM9 [54], TRIM67 [55]), the regulation of TAK1/IKK (TRIM45 [10]), and a direct influence on NF-κB transcriptional activity (TRIM21 [56]).

### 4.2. NLRP3 Inflammasome

The NLRP3 inflammasome pathway becomes activated in microglia and astrocytes by pathogens, cellular stress, or pathological protein aggregates such as β-amyloid and α-synuclein (α-Syn). NLRP3 activation occurs through two sequential stages, as follows: the priming phase—signals from TLRs or cytokines receptors (TNFR1, IL1R1) initiate NF-κB-dependent transcription of NLRP3, pro-IL-1β, and pro-IL-18; activation phase—DAMPs trigger NLRP3 oligomerization via the NEK7 protein, recruiting the adaptor ASC and procaspase-1 to form the functional inflammasome complex. Active caspase-1 then processes pro-IL-1β and pro-IL-18 into their bioactive forms, amplifying inflammatory response. In neurodegenerative conditions like AD and PD, chronic NLRP3 activation sustains IL-1β/IL-18 release and pyroptotic cell death, accelerating neuroinflammation and neuronal degeneration [57]. Several TRIMs affect NLRP3 inflammasome at different points. TRIM31 functions as an E3 ligase, targeting NLRP3 for K48-linked ubiquitination and proteasomal degradation to limit inflammasome assembly [13]. TRIM20 modulates inflammasome activity through interactions with different NLRP3 components (ASC, caspase-1, pro-IL-1β) [23,58]. TRIM14 suppresses NLRP3 activation by enhancing mitophagy in microglia [59].

**Figure 1:**
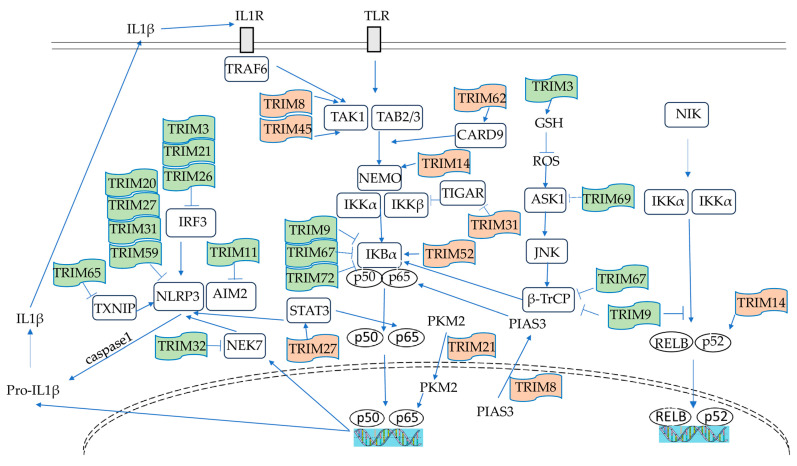
NF-κB and NLRP3 modulation by TRIM proteins. Only those mechanisms are presented for which there are published data on the specific target affected by the TRIM protein. The orange color reflects pro-inflammatory outcomes of TRIM function, the green color shows anti-inflammatory properties. Straight arrows denote activation, while T-shaped arrows denote inhibition.

NF-κB and NLRP3 have functional interdependence; NF-κB drives NLRP3 and pro-IL-1β transcription, and at the same time restrains excessive NLRP3 activity through p62-mediated mitophagy [60]. The IL-1β released in activated inflammasome enhances NF-κB via IL1R signaling. Figure 1 summarizes the available data from the literature on the specific mechanisms underlying NF-κB and NLRP3 modulation by TRIM proteins.

### 4.3. Interferon Pathways (cGAS/STING)

Type I IFNs play a dual role in inflammation, balancing antiviral defense while also potentially impacting chronic inflammation. The DNA sensor cGAS is crucial for initiating antiviral immune responses. This leads to the production of IFN-β, which enhances neuroinflammation through JAK-STAT signaling, and activates the expression of interferon-stimulated genes.

TRIM14, which is upregulated by type I IFN, stabilizes cGAS by recruiting USP14 to remove K48-linked ubiquitin chains, preventing cGAS degradation via autophagy and boosting IFN-I production in response to cytosolic DNA [41]. TRIM38 mediates the Small Ubiquitin-like Modification (SUMOylation) of cGAS (at K217/K464) and STING (at K338), stabilizing both proteins and enhancing immune signaling [42]. TRIM21 promotes the K48-linked ubiquitination of IRF3 [48] and IRF7 [61], as well as TRIM26 K48-polyubiquitinates IRF3 [49], targeting them for proteasomal degradation. This mechanism helps to prevent excessive IFN-I production but can also weaken antiviral defense. TRIM41 has been shown to catalyze the mono-ubiquitination of cGAS during herpes simplex virus 1 infection [62]. TRIM56 induces the K63-linked ubiquitination of STING at K150, promoting STING dimerization and TBK1 recruitment [51]. It also facilitates cGAS dimerization through mono-ubiquitination at K335 and STING at K150, enhancing their DNA-binding activity [31].

Although most of these mechanisms have been studied in non-neuronal cells during viral infection, evidence suggests that cGAS/STING signaling is also a driver of neuroinflammation in neurodegenerative diseases [63,64]. Aggregates of β-amyloid and α-Syn act as DAMPs, activating microglial cGAS/STING and TLR pathways. IFN-β drives microglia toward a pro-inflammatory phenotype, increasing the secretion of IL-1β and TNF-α while reducing anti-inflammatory cytokines. Postmortem analyses of brains from patients with PD and AD reveal upregulated IFN-I signaling, which may result from impaired mitophagy leading to the release of mitochondrial DNA into the cytosol and the subsequent activation of cGAS/STING [65].

Importantly, cGAS/STING signaling is closely related to NF-κB, since upon activation, STING binds to TBK1, which phosphorylates IRF3, on the one hand, and activates IKK, leading to the nuclear translocation of NF-κB subunits, on the other hand [66].

### 4.4. Regulation of Misfolded Proteins

Accumulated pathological aggregates (tau, α-Syn, superoxide dismutase 1), which are characteristic of neurodegeneration, act as DAMPs and promote inflammatory responses in the brain tissue. TRIM proteins employ diverse strategies—chaperoning, ubiquitination, SUMOylation, and autophagy induction—to manage misfolded proteins, thus helping to directly modulate neuroinflammation. For example, TRIM11 increases the proteasomal degradation of mutant tau via SUMOylation, as well as enhancing the solubility of normal tau, preventing its misfolding in AD [67]. In proteotoxic stress, TRIM16 upregulates genes in the ubiquitin pathway and p62 through NRF2 activation, and promotes the ubiquitination of misfolded proteins [24]. In neuronal cells, TRIM41 and TRIM17 exert opposing effects on transcription factor ZSCAN21 to regulate and control *SNCA* gene expression [32]. TRIM19 activates the clearance of nuclear protein aggregates implicated in neurodegenerative processes [68]. Conversely, TRIM28 facilitates the nuclear accumulation of aggregated proteins through its E3 ubiquitin ligase function, contributing to the development of PD and AD [47]. The ways TRIMs are able to modulate proteostasis are comprehensively reviewed in [5].

### 4.5. Modulation of the Transcriptional Activity of Genes Involved in Inflammatory Response

Several TRIM proteins function as epigenetic regulators of inflammatory gene expression via modulating chromatin or degrading transcriptional factors. For example, TRIM14 stabilizes the histone demethylase KDM4D by preventing its autophagic degradation, thereby reducing histone H3K9 trimethylation and promoting the transcription of pro-inflammatory cytokines such as IL-12 and IL-23 in microglia and brain tissue [33]. TRIM37 acts in microglia to ubiquitinate and degrade PPARγ, a transcription factor involved in anti-inflammatory responses, thus enhancing *IL-1β* expression and apoptosis during intracerebral hemorrhage [69]. Another example is TRIM24, whose targets for repression include such genes that are important to inflammation as *COX2* and *TNFAIP3* [34].

Importantly, not only the structural features of TRIM protein and compartmentalization determine its involvement in specific signaling pathways of the immune system and the role in inflammation in general. The same TRIM protein may have different functions depending on the type of stimulus, cell identity, or disease state.

Overall, the signaling pathways involving TRIM proteins are the same in both neuroinflammation and inflammation outside the brain. The question is whether specific mechanisms have emerged in brain cells, or whether TRIM activity depends solely on cell types, with the mechanisms remaining the same.

Next, we would like to consider the involvement of TRIM proteins in the immune response specifically within brain tissue. Since TRIMs are shown to be expressed in all major brain cell types (microglia, neurons, and astrocytes), it is of particular interest to examine their activity in neuroinflammation from the perspective of cell-type specificity.

## 5. TRIM Proteins Activity in Neuroinflammation

TRIM proteins can have either pro- or anti-inflammatory functions. However, they appear to form a complex regulatory network with considerable functional diversity across all the cell types.

To the best of our knowledge, this chapter represents most of the existing evidence on the action of TRIM proteins in the context of neuroinflammation studied in specific cell types of the brain tissue. The information is summarized in Table 2.

### 5.1. Microglia

Microglia are the main cells with immune functions in the CNS. Normally, microglia monitor the surrounding environment, and only activate when homeostasis in the environment changes. Microglial activation is initiated by pattern recognition receptors, which have specific affinities to pathogen-associated molecular patterns (PAMPs) such as components of bacterial, viral or fungal origin, and damage-associated molecular patterns (DAMPs) such as debris of damaged cells [70]. Microglial phenotype changes lead to two kinds of polarization states: M1 (pro-inflammatory) and M2 (anti-inflammatory). The activation of microglia is considered as the first sign of an inflammatory process in the CNS [71].

According to data from the literature, TRIMs function in microglia during neuroinflammation, contributing to it in different ways.

TRIM8 can enhance inflammation and apoptosis through TNF-induced NF-κB signaling in mice with cerebral ischemia–reperfusion (I/R) injury [72]. Moreover, TRIM8 contributes to NF-κB signaling in microglial cells in a model of oxygen-glucose deprivation (OGD), and this effect is attenuated by miR-665-3p, which is known as an inflammation-associated miRNA [73].

TRIM20 functions as a regulator of the crosstalk between autophagy and inflammation in microglial cells and macrophages, serving as a link between autophagy and the NLRP3 inflammasome in microglia and infiltrating monocytes. It serves as an adaptor for inflammasome components NLRP3, NLRP1, and proCASP1, and promotes their degradation by autophagy in the context of traumatic brain injury (TBI) [74].

TRIM21 promotes neuroinflammation through the M1 polarization of microglia, while its knockdown suppresses the M1 polarization and negative effects caused by the NF-κB/NLRP3 inflammasome pathway [56]. In microglia infected by Japanese encephalitis virus, TRIM21 was described to negatively regulate the production of p-IRF3 and IFN-β [75].

TRIM27 is increased in a hypoxic–ischemic encephalopathy model, while its downregulation reduces the brain infarct volume and the production of inflammatory factors, increases the quantity of M2, and decreases the quantity of M1 microglia cells by regulating the STAT3/HMGB1 axis [76]. However, another study has shown that TRIM27, on the contrary, acts in a neuroprotective way in a mouse ischemic stroke model by suppressing the activation of NLRP3 inflammasome [77].

TRIM28 is acknowledged as the principal E3 ubiquitin ligase responsible for HK2 ubiquitination in microglial cells, which decreases its degradation mediated by the immune checkpoint VISTA, thus increasing microglia glycolysis and activation in the murine sepsis model [78]. Moreover, TRIM28 is the central orchestrator of ferroptosis in microglia, a cell death mechanism characterized by the accumulation of iron ions, which leads to iron toxicity, oxidative stress, and inflammation. By suppressing GSK3B expression, TRIM28 attenuates autophagy but promotes ferroptosis, leading to ferroptosis-induced neuroinflammation and neuropathic pain [79].

TRIM31 is the downstream factor contributing to the anti-inflammatory therapeutic effect of parthenolide. In microglia, parthenolide drastically decreases the levels of pro-inflammatory cytokines and increases IL-10 release. It also decreases reactive oxygen species (ROS) and restores microglial phagocytic activities via the AKT/MAPK/NF-κB signaling pathway. This action was attributed to the upregulated expression of TRIM31, which directly interacts with the NLRP3 inflammasome, promoting its ubiquitination and degradation [80]. An increase in TRIM31 was observed after Modified Danzhi Xiaoyaosan (MDZXYS) treatment in rats with depression, along with a decrease in the expression of NLRP3 inflammasome-associated proteins and a reduction in depressive behavior [81].

TRIM32 acts as a positive factor in the context of spinal cord injury (SCI), as its deficiency impairs axonal regeneration, and leads to the increased proliferation of astrocytes and microglia and the production of inflammation-linked cytokines [82]. Its level increases after SCI, and it suppresses microglia pyroptosis through the downregulation of NEK7 [83]. However, in meningitis caused by *Streptococcus suis*, TRIM32 deficiency is proven to decrease the production of inflammatory cytokines [84].

TRIM37 mRNA is increased in the peripheral blood mononuclear cells of patients with intracerebral hemorrhage. TRIM37 regulates the ubiquitination of PPARγ, which is a neuroprotective receptor, and thus promotes thrombin-stimulated apoptosis and IL-1β release both in the mouse microglia cell line [69] and in the rat brain after middle cerebral artery occlusion and reperfusion (MCAO) [85]. The knockdown of *TRIM37* reduces brain damage [85].

TRIM45 is upregulated in the peri-infarct area of mice exposed to cerebral I/R injury. Its functions are pro-inflammatory, performed via the polyubiquitination of TAB2. That leads to the formation of the TAB1-TAK1-TAB2 complex, followed by the activation of TAK1, and, consequently, the activation of the NF-κB pathway [10]. However, TRIM45 promotes neuroinflammation not only through NF-κB signaling, but also through microglia pyroptosis via the Atg5/NLRP3 axis [86].

TRIM52 activates the NF-κB pathway via promoting IκBα ubiquitination in LPS-treated rat microglial cells [87].

TRIM59 mediates the ubiquitination of NLRP3 in microglia upon brain I/R injury, thus degrading NLRP3 and alleviating the injury [88].

*TRIM62* expression is elevated in microglia treated with OGD, whereas its knockout alleviates neuroinflammation in cerebral ischemia. Under the conditions of I/R, TRIM62 undergoes K63-mediated ubiquitination, which enables its interaction with NLRP3 [89]. These results are in line with the detrimental effects of TRIM62 in various murine models of diabetes, where it activates the TLR4/NFkB and NLRP3 inflammasome pathways in the hippocampus [90].

TRIM67 alleviates inflammation in the brain after middle cerebral artery occlusion/reperfusion (MCAO/R) in mice and in primary cultured microglia subjected to OGD/R by reduced K48-linked ubiquitination and the increased K63-linked ubiquitination of IκBα, which leads to the suppression of NF-κB signaling [55].

TRIM69 protects against inflammation and apoptosis in high-fat diet-treated mice and in LPS-treated microglia cells via ASK-1 inactivation [91].

### 5.2. Astrocytes

Under normal conditions, astrocytes perform a variety of functions, including the maintenance of the blood–brain barrier (BBB), synapse formation and support, the regulation of cerebral homeostasis, and blood flow [92]. In pathological processes characterized by the activation of microglia, astrocytes become reactive, secreting increased amounts of pro-inflammatory cytokines (TNF-α, IL-1β, IL-6, etc.). It has been proven that, as well as microglial cells, astrocytes can reach pro-inflammatory and anti-inflammatory states of activation, named A1 and A2, respectively. The role of astroglia in various neuroinflammatory diseases is currently widely studied [93], although the information on the role of TRIMs in astrocytes in neuroinflammation is limited. Recently, we showed that a number of *TRIM* genes change their expression in glial and neuronal cultures derived from the induced pluripotent stem cells of patients with PD in comparison with corresponding cell lines from healthy donors exposed to TNF-α [94]. The expressions of most studied *TRIM* genes (*TRIM2*, 4, 5, 9, 16, 22, 24, 32, 34, 38, 47, 66 and 69) were lower in glial cells of PD patients compared to healthy donors, and remained lower, or in some cases even underwent further downregulation, in the presence of TNF-α. *TRIM6* and *TRIM36* served as exceptions, being upregulated in the glia of PD patients. However, in the glia from healthy donors, TNF-α upregulated the expression of a number of *TRIM* genes. It should be noted that the most pronounced changes were detected in the glial cultures [94]. However, the published evidence on the role of individual TRIM proteins in modulating the inflammatory response in glial cells is scarce.

TRIM8 activates apoptosis and NF-κB signaling in astrocytes as well as microglia, exacerbating inflammation in LPS-induced astrocytes [72].

TRIM21, proven to have pro-inflammatory effects in the context of microglial polarization, was recently discovered to be one of the more important examples of the inflammation-related action of TRIM proteins in astrocytes. In experimental autoimmune encephalomyelitis (EAE), an in vivo model of multiple sclerosis, TRIM21 contributes to astrogliosis by promoting the nuclear localization of the glycolytic enzyme PKM2, while its knockdown decreases experimental EAE [44].

### 5.3. Neurons

Neurons play a critical role in initiating and propagating neuroinflammation in the brain through their interactions with microglia and the release of various signaling molecules. Under neuroinflammatory conditions, the damaged and dying neurons produce large amounts of DAMPs, contributing to microglia activation. When activated, microglia release pro-inflammatory cytokines and toxic products, such as ROS and nitric oxide, which further harm neurons and amplify the positive feedback loop [3]. Glutamate, HMGB1, and nucleotides are among the molecules released by damaged neurons that lead to microglia activation [95]. In neurodegenerative disorders associated with pathological protein aggregation in neurons, such as AD, PD, and amyotrophic lateral sclerosis, protein aggregates exit the dying neurons and activate microglia. However, if the clearance of the aggregates fails, microglia become chronically activated, boosting neuronal damage and protein accumulation. According to the latest proceedings, neuronal loss appears in such diseases much earlier than protein aggregation, which explains why the treatments aimed only at the aggregates fail to stop those diseases [3]. However, neurons, both healthy and damaged, are able not only to activate microglia, but also to suppress it by producing anti-inflammatory cytokines such as fractalkine and TGF-β [95]. Finally, neurons take part in neuroinflammation not only through interactions with microglia, but also independently through the activation of the inflammasome. Specifically, the NLRP1 inflammasome in neurons is activated by amyloid-β in AD, leading to caspase-1 activation, which triggers inflammation and neuronal apoptosis [96]. Thus, neurons are not only victims of neuroinflammation, but are an important part of the game. TRIMs’ functions in neurons in the context of neuroinflammation are multifaceted.

TRIM3 is significantly decreased in mice MPTP models of PD. *TRIM3* mRNA is lower in the venous blood of PD patients. In SH-SY5Y cells induced by an MPP+ solution, TRIM3 reduces apoptosis, elevating the expression of the Bcl-2 protein and downregulating the expression of Bax, Cleaved-caspase 3 and Cleaved-caspase 9 proteins, and also increasing glutathione and superoxide dismutase levels while reducing ROS [97].

TRIM6 is identified as a driving factor in the PD mouse model, promoting higher levels of α-Syn and neuronal damage [98].

TRIM9, which is a brain-specific TRIM protein, resists neuroinflammation and neuronal death via the negative regulation of the NF-κB-dependent inflammatory reaction in a mouse ischemic stroke model [54]. TRIM9 is repressed in the brains of patients suffering PD and dementia with Lewy bodies [40]. It is upregulated in the brain of rats that have undergone treadmill exercise, showing a protective effect in the streptozocin-induced model of AD due to the inhibition of NF-κB signaling [99]. TRIM9 binds to the WD40 repeat region of β-TrCP, thus impairing β-TrCP interaction with IκBα and p100, thereby leading to a decrease in both canonical and non-canonical NF-κB activation [17].

TRIM11 plays an important role in AD prevention and is downregulated in AD brains. It acts as a molecular chaperon for tau, enhances its solubility, and promotes the degradation of both misfolded and normal tau proteins. Furthermore, TRIM11 has beneficial effects when administrated to the hippocampi of PS19 and 3xTg-AD mice. TRIM11 binds to and SUMOylates tau, especially the mutant form, promoting its degradation in the proteasome. Moreover, it enhances neuronal integrity and synaptic formation [100].

TRIM13 is known to act in an anti-inflammatory way in the CNS. Thus, the brain-specific deletion of *TRIM13* aggravates high-fat-diet-induced neuroinflammation in mice, leading to an increase in the levels of pro-inflammatory cytokines and NF-κB activation [101].

TRIM14 is increased in a rat model of cerebral I/R, and *TRIM14* inhibition by siRNA leads to an anti-inflammatory effect due to NF-κB/NLRP3 pathway activation [59].

TRIM21 effectively neutralizes antibody-labeled tau assemblies in the human neuronal cell line SH-SY5Y [102].

TRIM22 is found to be increased in HCN-2 neurons treated with OGD/reoxygenation and in ischemic cortex tissues from MCAO/R mice. Its knockdown acts anti-inflammatorily due to the inhibition of the NF-κB/NLRP3 axis [103].

TRIM24 is the downstream factor involved in the action of esketamine, improving cognitive dysfunction in AD. Esketamine increased PI3K and AKT phosphorylation in the hippocampus of triple transgenic AD (3xTg-AD) mice, but its action has been reversed by *TRIM24* knockdown [104].

TRIM28 regulates α-Syn and tau in neurons by promoting their nuclear localization and accumulation, which is shown in mouse models of synucleinopathy and tauopathy [47].

TRIM29 is overexpressed in MCAO murine models of stroke and the OGD cell model, while its loss aggravates inflammation in both models. It directly interacts with NLRC4 and promotes its K48-linked polyubiquitination, as well as proteasomal degradation [105].

TRIM31 is downregulated in ischemic brains, and its deficiency alleviates the brain damage caused by MCAO. The mechanism behind this action is that TRIM31 promotes the polyubiquitination of TIGAR and, consequentially, its proteasomal degradation. TIGAR is predominantly expressed in neurons, where it protects against brain ischemia by increasing the pentose phosphate pathway flux and preserving mitochondria function [106].

TRIM32 sensitizes SH-SY5Y dopaminergic neuronal cells to apoptosis by translocating to the mitochondria and localizing to the outer mitochondrial membrane under PD stress conditions. The protein regulates mitochondrial function since it decreases complex I activity, upregulates cellular ROS levels, and decreases cellular ATP levels. TRIM32 also promotes neuronal death by reducing the level of XIAP [38]. *TRIM32* knockdown promotes the survival of primary hippocampal neurons under the conditions of OGD/reoxygenation by activating the NRF2 signaling pathway [107].

TRIM44 contributes to inflammation upon TBI being significantly increased along with TLR4/NF-κB activation, whereas its silencing inhibits inflammation and alleviates the condition after TBI [108].

TRIM47 is found to be important in the context of neuroinflammation induced by cerebral I/R injury. Its reduction has been associated with a decrease in infarct size, the mitigation of neurological deficits scores, as well as the suppression of apoptosis through inhibiting caspase-3 cleavage, and a decreased release of pro-inflammatory factors. These effects are associated with NF-κB pathway suppression [109]. *TRIM47* knockdown is also proven to ameliorate inflammation, neuronal cell apoptosis, neurological damage, and cognitive deficits induced by sevoflurane in rats via the inhibition of the NF-κB pathway, which is in line with the data obtained for the I/R injury model [110]. However, recent studies have shown that TRIM47 can stimulate the NRF2 pathway in human brain endothelial cells, thereby regulating BBB permeability, protecting brain tissue from oxidative damage, and supporting cognitive function. Its protective effects have been demonstrated in cerebral small vessel disease, a leading contributor to stroke, cognitive decline, and dementia [111]. Collectively, these findings suggest that TRIM47 has a context-dependent dual function in neuroinflammation; it may enhance the protective NRF2 response or, under certain conditions, facilitate pro-inflammatory NF-κB signaling.

TRIM56 plays a beneficial role after SCI, controlling PANoptosis, a newly discovered type of pro-inflammatory cell death accompanied by the formation of PANoptosome, a multiprotein complex that simultaneously regulates pyroptosis, apoptosis, and necroptosis. TRIM56 promotes the ubiquitination and degradation of YBX1, and therefore inhibits *ZBP1* expression and alleviates PANoptosis in the pathogenesis of SCI [112].

TRIM65 possesses neuroprotective functions due to its ability to regulate NLRP3. The conjugation of TRIM65 to NLRP3 has been shown to be protective in CNS inflammation induced by the MCAO model of stroke and in the CUMS model of depression via NLRP3 inflammasome suppression [113,114].

TRIM67 decreases inflammation in cerebral I/R injury, primary cultured microglia, and in the hypothalamus of obese mice, inhibiting NF-κB signaling [55,115].

TRIM72 possesses the ability to block LPS-induced neuroinflammation due to different mechanisms, such as inhibiting the TLR4/NF-κB pathway [116] and NLRP3/Caspase-1/IL-1β axis [117]. It also ameliorates inflammation, as well as neuropathic pain and oxidative stress, in the context of chronic constriction injury through the activation of NRF2/HO-1 signaling [118]. Recombinant human TRIM72 opposes inflammation in I/R brain injury by inhibiting apoptosis and enhancing pro-survival RISK signaling [119]. In mouse brain tissues and primary rat cortex neurons treated with Aβ42 or cerebrospinal fluid from AD patients, recombinant TRIM72 increases plasma membrane repair capacity, normalizing ROS [36].

**Figure 2:**
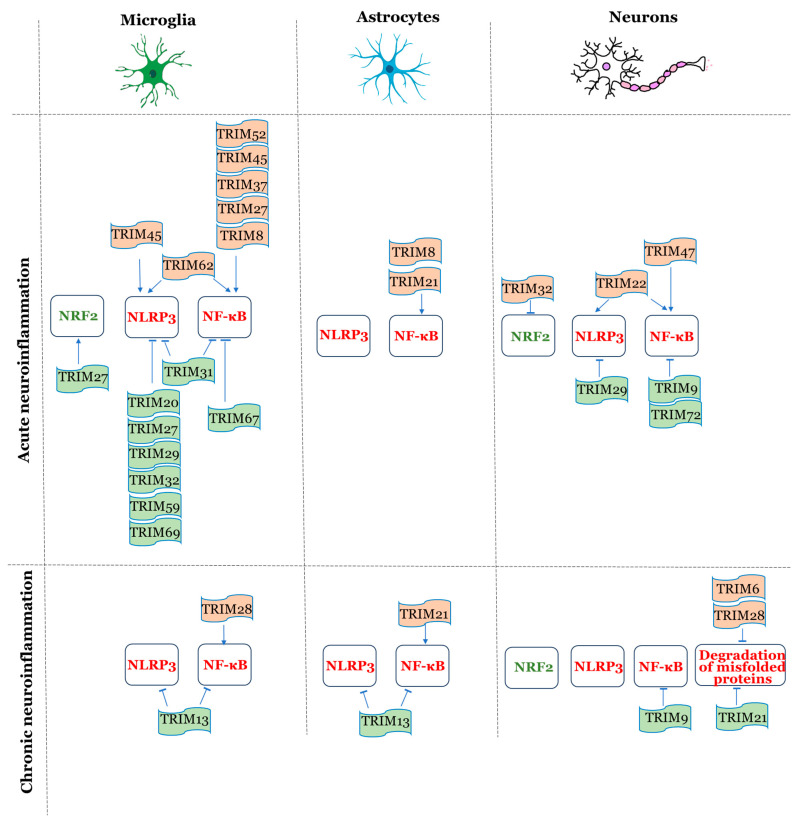
Existing evidence on the TRIM protein modulation of key inflammatory signaling pathways, specifically in microglia, astrocytes, and neurons. Red and orange colors reflect pro-inflammatory outcomes of TRIM function, while green represents anti-inflammatory properties. Straight arrows denote activation, while T-shaped arrows denote inhibition.

A significant number of studies on the involvement of TRIM proteins in neuroinflammation have revealed several mechanisms, but consider them in general, without taking into account the specific cell types present in brain tissue. Figure 2 consolidates the current evidence—to the best of our knowledge—regarding the TRIM protein modulation of key inflammatory signaling pathways studied specifically in microglia, astrocytes, and neurons.

To sum it up, most of the available information is related to microglia. This may be partly because microglia are the primary immune cells in brain tissue, but it also reflects the fact that this cell type has been the most extensively studied in this context. Further research is needed to expand our understanding of these processes in neurons and astrocytes.

**Table 2:** Published data on the role of TRIM proteins in neuroinflammation.

Condition/ Pathology	TRIM Protein	Directionality		Expression Level vs. Normal	Mechanism/ Pathway	Target Cells	References
Acute neuroinflammation							
Acute injury (I/R, SCI, TBI)	TRIM8	Pro-inflammatory		↑	TRIM8 knockdown or miR-665-3p overexpression blocks the OGD-induced activation of NF-κB signaling	Microglia, mice	[73]
		Pro-inflammatory		↑	Activates NF-κB signaling	Brain tissue and astrocytes, mice	[72]
	TRIM9	Anti-inflammatory		↑	Suppresses NF-κB signaling	Neurons, mice	[54]
	TRIM14	Pro-inflammatory		↑	Activates NF-κB/NLRP3 signaling	Brain tissue, rats	[59]
	TRIM20	Anti-inflammatory		↑	Targets inflammasome components (NLRP3, NLRP1, and proCASP1) for autophagic degradation	Microglia, mice	[74]
	TRIM22	Pro-inflammatory		↑	Activates NF-κB/NLRP3 signaling axis	Neurons, mice	[103]
	TRIM27	Anti-inflammatory		↓	Suppresses NLRP3 and activates the AKT/NRF2/HO-1 pathway	Microglia, mice	[77]
		Pro-inflammatory		↑	Increases inflammation and microglia cell activation via STAT3/HMGB1 axis	Microglia, mice	[76]
	TRIM29	Anti-inflammatory		↑	Ubiquitinates NLRC4, attenuating pro-inflammatory mediators’ production	Neurons, microglia, mice	[105]
	TRIM31	Pro-inflammatory		↑	Induces ROS and inflammation via TIGAR ubiquitination	Neurons, rats	[106]
	TRIM32	Anti-inflammatory		↑	Drives axonal regeneration and increases the proliferation of astrocytes and microglia	Astrocytes, microglia, mice	[82]
				↑	Suppresses microglia pyroptosis through downregulation of NEK7	Microglia, mice	[83]
		Pro-inflammatory		↑	Suppresses the NRF2 signaling pathway	Neurons, mice	[38]
	TRIM37	Pro-inflammatory		↑	Promotes PPARγ ubiquitination, increasing IL-1β release	Microglia, mice	[69]
	TRIM44	Pro-inflammatory		↑	Activates TLR4/NF-κB signaling	Brain tissue, rats	[108]
	TRIM45	Pro-inflammatory		↑	Activates NF-κB signaling	Microglia, mice	[10]
	TRIM47	Pro-inflammatory		↑	Activates NF-κB signaling	Neurons, rats	[109,110]
	TRIM56	Anti-inflammatory		↓	Ubiquitinates YBX1 which ameliorates ZBP1-mediated neuronal PANoptosis	Neurons, mice	[112]
	TRIM59	Anti-inflammatory		↓	Inhibits NLRP3	Microglia, human HMC3 cells	[88]
	TRIM62	Pro-inflammatory		↑	Activates NLRP3 and NF-κB signaling	Microglia, mice	[89]
	TRIM65	Anti-inflammatory		↓	Competes with TXNIP for binding to NLRP3 inflammasome and downregulates it	Brain tissue, mice	[113]
	TRIM67	Anti-inflammatory		↓	Promotes IκBα stability and inhibits NF-κB activity	Microglia, brain tissue, mice	[55,115]
	TRIM72	Anti-inflammatory			Crosses the BBB, protects injury through activation of the RISK signaling pathway	Brain tissue, neuronal stem cells, rats	[119]
		Anti-inflammatory			Activates NRF2/HO-1 signaling ameliorating inflammation, neuropathic pain, and oxidative stress	Spinal cord, rats	[118]
Acute brain infections	Bacterial	TRIM8	Pro-inflammatory	↑	Activates NF-κB signaling	Astrocytes, mice	[72]
		TRIM28	Pro-inflammatory	↑	Ubiquitinates HK2, knockdown of TRIM28 reduces the pro-inflammatory response	Microglia, mice	[78]
		TRIM31	Anti-inflammatory		Inhibits NLRP3 signaling	Microglia, mouse BV2 and human HMC3 cells	[80]
		TRIM32	Pro- inflammatory		Increases the production of pro-inflammatory cytokines and chemokines,	Brain tissue, mice	[84]
			Anti-inflammatory		Decreases the recruitment of polymorphonuclear neutrophils and macrophages in early infection	Brain tissue, mice	[84]
		TRIM45	Pro- inflammatory	↑	Activates NLRP3 by altering Atg5 and regulating autophagic flux, increases pyroptosis	Microglia, mice	[86]
		TRIM52	Pro- inflammatory	↑	Enhances NF-κB signaling by promoting IkBa ubiquitination	Microglia, rats	[87]
		TRIM69	Anti-inflammatory	↑	Attenuates the activation of ASK1 and NF-κB, along with the subsequent down-regulation of inflammatory cytokines	Microglia, mice	[91]
		TRIM72	Anti-inflammatory		Reduces TLF4/NF-κB signaling	Neurons, mice	[116]
			Anti-inflammatory	↑	Combined with hUC-MSCs, inhibits the NLRP3/caspase-1/IL-1β axis	Brain tissue, mice	[117]
	Viral	TRIM21	Pro-inflammatory	↑	Negatively regulates IFN-β production mediated by IRF-3	Microglia, human CHME3 cells	[75]
Chronic neuroinflammation							
Neurodegenerative diseases		TRIM3	Anti-inflammatory	↓	Increases GSH and SOD levels, reduces ROS	Neurons, mice	[97]
		TRIM6	Pro-inflammatory		Promotes higher levels of α-Syn	Neurons, mice	[98]
		TRIM9	Anti-inflammatory	↓	Inhibits NF-κB signaling	Brain tissue, rats	[99]
		TRIM11	Anti-inflammatory	↓	Enhances tau solubility, promotes the proteasomal degradation of mutant tau	Neurons, human postmortem	[100]
		TRIM21	Anti-inflammatory		Neutralizes tau seeding	Neuronal human SHSY-5Y cells	[102]
			Pro-inflammatory	↑	Promotes PKM2 nuclear translocation and astrocyte activation	Astrocytes, mice	[44]
		TRIM24	Anti-inflammatory		Activates the downstream PI3K/AKT signaling	Brain tissue, mice	[104]
		TRIM28	Pro-inflammatory	↑	Promotes accumulation of α-synuclein and tau	Brain tissues, mice, human post-mortem	[47]
		TRIM32	Pro-inflammatory		Decreases complex I activity on mitochondria and, subsequently, increases cellular ROS levels	Neuronal human SHSY-5Y cells	[107]
		TRIM72	Anti-inflammatory		Increases plasma membrane repair capacity, normalizing ROS	Brain tissue, mice; neurons, rats	[36]
CUMS depression		TRIM31	Anti-inflammatory	↓	Negatively regulates the NLRP3 inflammasome	Brain tissue, mice	[81]
		TRIM65	Anti-inflammatory	↓	Inhibits NLRP3 inflammasome assembly, caspase-1 activation and IL-1β secretion	Brain tissue, mice	[114]
High-fat-diet/obesity-induced neuroinflammation		TRIM13	Anti-inflammatory	↓	Decreases NF-κB signaling pathway	Brain tissue, mice	[101]
		TRIM62	Pro-inflammatory		Activates the TLR4/NF-κB pathway and NLRP3 inflammasome	Brain tissue, mice	[90]
		TRIM67	Anti-inflammatory	↓	Decreases NF-κB signaling pathway, increases BDNF expression	Brain tissue, mice	[115]
		TRIM69	Anti-inflammatory	↓	Decreases NF-κB signaling pathway and activation of ASK1	Brain tissue, mice	[91]
Neuropathic pain		TRIM28	Pro-inflammatory		Suppresses GSK3B, increases ferroptosis	Microglia, mice	[79]

↑ symbol reflects an increased level of expression of the TRIM protein in a certain condition/pathology; ↓ symbol reflects a decreased level of expression.

## 6. The Role of TRIM Proteins in Acute and Chronic Neuroinflammation

TRIM proteins are “molecular switches” whose functions are determined not only by the cell type, but by the phase of neuroinflammation and pathological context [8].

Neuroinflammation can manifest as either acute or chronic inflammation. Acute neuroinflammation involves the activation of resident immunocompetent cells (microglia and astrocytes), the recruitment of peripheral immune cells, and the release of pro-inflammatory cytokines. These events are aimed at eliminating damaging agents and initiating tissue repair. However, excessive or unresolved acute inflammation can lead to secondary tissue damage and worsen neurological outcomes. In contrast, chronic neuroinflammation is characterized by the sustained activation of immune cells and the prolonged release of inflammatory mediators. It is commonly linked to neurodegenerative diseases such as AD, PD, and amyotrophic lateral sclerosis, as well as to aging, autoimmunity, and persistent exposure to toxic metabolites or environmental insults. Chronic inflammation contributes to progressive neuronal dysfunction and loss, often exacerbated by ongoing protein aggregation and impaired clearance mechanisms [120,121].

Table 2 summarizes current evidence on the roles of TRIM family proteins in neuroinflammation, categorizing the data according to three primary causes, namely, acute injury, acute infection, and chronic neuroinflammation, which are characteristic of different pathologies.

The information given in Table 2 demonstrates that both pro-inflammatory and anti-inflammatory TRIM proteins are upregulated during acute inflammation. Pro-inflammatory proteins enhance NF-κB and NLRP3 inflammasome signaling (TRIM8 [72], TRIM14 [59], TRIM22 [103], TRIM32 [84], TRIM44 [108], TRIM45 [10], TRIM47 [109,110], TRIM52 [87], TRIM62 [89]). A number of anti-inflammatory TRIMs are also upregulated and suppress the excessive activation of NF-κB and inflammasome, promoting inflammation resolution and protecting neurons (TRIM9 [54], TRIM20 [74], TRIM29 [105], TRIM69 [91]). In this regard, acute inflammation requires an optimal temporal and quantitative balance between pro- and anti-inflammatory TRIMs.

In long-lasting inflammatory conditions, as in the case of neurodegeneration, TRIM proteins also play a dual role; some support pathological neuroinflammation, while others suppress it, but the balance between them is disrupted. Pro-inflammatory TRIM21 [44] is upregulated, while numerous anti-inflammatory ones (TRIM3 [97], TRIM9 [99], TRIM11 [100], TRIM13 [101]), TRIM65 [114], TRIM67 [115]) are suppressed. The dysregulation of TRIMs functions can contribute to the development of chronic inflammation [122].

## 7. TRIM Proteins in Neuroinflammation Versus Other Inflammatory Processes

**Figure 3:**
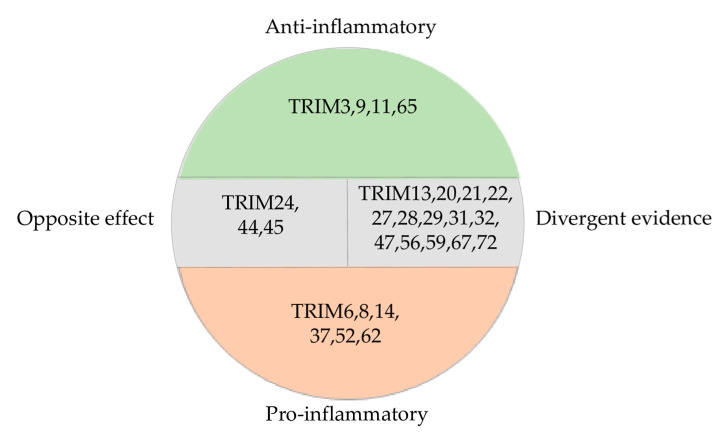
A comparative analysis of available evidence regarding the roles of TRIM proteins in inflammation in the CNS and beyond. Orange reflects TRIMs consistently recognized in the literature data as pro-inflammatory, green denotes TRIMs that are consistently recognized as anti-inflammatory, grey indicates TRIMs for which the evidence is conflicting (opposite) or divergent.

A vast majority of TRIM proteins that are involved in the regulation of CNS inflammation also participate in inflammation in other organs and tissues of the body. In this context, many TRIMs exhibit a similar functional orientation, either pro- or anti-inflammatory; however, this is not universally applicable to all members of the TRIM family. This section of the review aims to summarize and trace the correlation between known data on TRIM protein participation in neuroinflammation and in other inflammatory pathologies. Data for proteins with documented evidence in both contexts are visualized in Table 3 and compiled in Figure 3.

### 7.1. TRIMs with Pro-Inflammatory Functions

Pro-inflammatory TRIMs mainly act through the activation of NF-κB and NLRP3 signaling, although some of them are involved in additional mechanisms.

TRIM6 promotes higher levels of α-Syn in PD [98], and at the same time, it induces ROS and pyroptosis, inflicted by Ang II, in proximal tubule epithelial cell line HK2 via the ubiquitination of the GPX3 protein, leading to the upregulation of the NLRP3 inflammasome [126]. TRIM6 serves as an important regulator of antiviral immunity since it promotes IKKε oligomerization and autophosphorylation, leading to the formation of antiviral IFN-I signaling complexes [188].

TRIM8 was reported to have pro-inflammatory effects in CNS cells and tissues [72,73]. Along with functions in the context of CNS, knockdown led to the attenuation of inflammatory responses in osteoarthritis chondrocytes due to the inactivation of NF-κB signaling [127]. It enhances the NF-κB pathway by promoting the K63-ubiquitination of TAK1, and thus plays a pro-inflammatory role in *Pseudomonas aeruginosa*-induced keratitis [9] and in dextran sulfate sodium-induced colitis in mice [128]. The suppression of TRIM8 lessens the inflammatory response in an LPS-induced model of acute lung injury through the inactivation of NF-κB signaling and the promotion of the AMPKα pathway [189]. TRIM8 silencing upon allergic asthma blocks TAK1 and inhibits the NF-κB/NLRP3 pathway [129]. Interestingly, TRIM8 affects NF-κB signaling through several different mechanisms. Firstly, it induces PIAS3 translocation from the nucleus to the cytoplasm, which leaves p65 free to conduct its function. Secondly, in response to TNF-α, TRIM8 is translocated to the cytoplasm, where it ubiquitinates TAK1. Moreover, different TRIM8 domains (RING, CC, C-terminal) may regulate NF-κB in discrete ways [39].

TRIM14 activates the NF-κB pathway in the CNS [59]. It also participates in endothelial NF-κB activation through binding to NEMO and enhancing the phosphorylation of IκBα and p65 [134]. However, it is not the only way in which TRIM14 may regulate the NF-κB pathway [135,136]. TRIM14 influences inflammation not only via NF-κB regulation. It also acts as an epigenetic regulator by suppressing histone H3K9 trimethylation, preventing the histone demethylase KDM4D from undergoing degradation, which leads to the expression of the KDM4D-directed pro-inflammatory cytokines. This mechanism enables the protection of *TRIM14*-deficient mice against inflammation in the EAE model of multiple sclerosis [33].

TRIM37 is upregulated in patients with intracerebral hemorrhage and in rat models of brain damage, where it promotes PPARγ ubiquitination, leading to increased apoptosis and IL-1β release, with its knockdown reducing brain damage [69,85]. However, several studies on hepatic and pulmonary inflammatory pathology underline its role in NF-κB activation [166,167,168].

TRIM52 participates in NF-κB pathway activation in microglia [87]. Outside the CNS, TRIM52 aggravates LPS-induced inflammation through the TLR4/NF-κB pathway in human periodontal ligament cells [170] and sulfate sodium-induced inflammatory bowel disease in mice [171]. The same mechanism is relevant for IL-1β-treated synovial fibroblasts where TRIM52 promoted cell proliferation, inflammatory response, and oxidative stress via the TLR4/NF-κB signaling pathway [172]. Similarly, TRIM52 is a positive regulator of the NF-κB pathway in HEK293T cells [173].

TRIM62 enhances neuroinflammation through NLRP3 inflammasome interaction and the TLR4/NF-κB pathway [89,90]. In myocytes, *TRIM62* knockdown reduces the LPS-mediated increase in *IL-6* expression [180]. Moreover, TRIM62 K27-ubiquitinates CARD9, activating inflammatory response to fungal infection via NF-κB and MAPK signaling in the dextran sulfate sodium mice model of intestinal inflammation, and upon *C. albicans* infection [179]. More specifically, TRIM62 activates the TRIF branch of the TLR4 signaling pathway, and thus induces NF-κB and AP-1 activity [22].

### 7.2. TRIMs with Anti-Inflammatory Properties

Anti-inflammatory TRIMs act mainly, but not exclusively, through the suppression of NF-κB and/or NLRP3 activity.

TRIM3 increases glutathione and superoxide dismutase levels while reducing ROS in SH-SY5Y cells and the PD mouse model [97]. At the same time, it represses the IRF3 pathway and NLRP3 inflammasome activation via interacting with IRF3 and inhibiting its phosphorylation in rat kidney upon LPS treatment [123].

TRIM9’s anti-inflammatory properties, related to the inhibition of NF-κB, are studied in the context of neuroinflammation [17,54,99]. Similar results were obtained in TNF-α and IL-1β-stimulated human HEK293T and A549 cell lines [17].

TRIM11 is known as the protein that increases tau solubility, thus preventing AD [100]. However, its functions go beyond the regulation of tau. TRIM11 is a key regulator of the AIM2 inflammasome, which it suppresses upon DNA virus infection through facilitating AIM2–p62 interaction, leading to the delivery of TRIM11–AIM2 protein complexes to autophagosomes for degradation [130].

TRIM65 negatively regulates the NLRP3 inflammasome and has neuroprotective functions [113,114]. In vitro experiments have shown that TRIM65 suppresses NLRP3 inflammasome activation in various cell lines, directly interacting with NLRP3 and promoting NLRP3 K48- and K63-linked ubiquitination, and it was suggested that the NACHT domain of NLRP3 could be ubiquitinated by the RING domain of TRIM65. In line with these results, TRIM65 was discovered to alleviate multiple mouse models of NLRP3-dependent inflammatory diseases, such as LPS-induced systemic inflammation, MSU-induced peritonitis and gouty arthritis, by downregulating NLRP3 [15].

### 7.3. TRIMs with the Opposite Effects in the CNS and Other Organs and Tissues

TRIM24 has a protective function in AD enacted via the downstream PI3K/AKT pathway [104]. However, in macrophages, TRIM24 prevents polarization into the M2 phenotype and inhibits *IFNβ1* and *IL-10* transcription by suppressing the recruitment of CBP/p300, whereas its knockout promotes *IFNβ1* and *IL-10* expression and protects mice from LPS-induced endotoxic shock, which makes TRIM24 a potent pro-inflammatory factor in macrophages [147].

TRIM44 exacerbates inflammation upon TBI [108]. However, in renal I/R injury, the protein plays a protective role by inhibiting pyroptosis through inhibition of the NLRP3 inflammasome [169].

TRIM45 is upregulated in the peri-infarct area following cerebral ischemia, and promotes neuroinflammation through NF-κB activation and microglia pyroptosis [10,86]. There is, however, evidence obtained from experiments with human cell line HEK293 demonstrating that TRIM45 negatively regulates TNF-induced NF-κB-mediated transcriptional activity and cell proliferation [30].

### 7.4. TRIMs with Divergent Evidence

The available data on several TRIM proteins are conflicting, as they demonstrate both pro-inflammatory and anti-inflammatory effects in neuroinflammation and/or other types of inflammation.

TRIM13 has anti-inflammatory effects in the CNS [101]. At the same time, TRIM13 suppresses the TNF-induced NF-κB immune response in human cell cultures, such as HEK293, HeLa, A549 and MCF7, by modulating the activity of NEMO, which regulates the assembly of the IKK complex [131]. However, under some conditions, TRIM13 possesses pro-inflammatory activities. For instance, it takes part in SARS-CoV-2 nonstructural protein 6 (NSP6)-induced NF-κB activation by linking polyubiquitin chains to NSP6, followed by the recruitment of NEMO to the NSP6–TAK1 complex [133]. Interestingly, it also mediates the ubiquitination of Nur77, a nuclear receptor that regulates inflammation. The ubiquitination and degradation of Nur77 leads to TNF-α-mediated IL-6 production [132].

TRIM20 is associated with high autophagy flux in microglia, which is generally considered to reduce inflammation. Specifically, it targets inflammasome components, such as NLRP3, NLRP1, and proCASP1, for autophagic degradation [74]. In the same way, in macrophages, TRIM20 forms a complex with autophagy components ULK1, BECN1, and ATG16L1, and promotes the autophagic degradation of NLRP3, NLRP1, and proCASP1 [138]. However, in blood neutrophils, TRIM20 interacts with β2-microglobulin to promote “pyrin inflammasome” formation and the secretion of IL-1β [137]. However, in macrophages, it targets inflammasome components (NLRP3, NLRP1, and proCASP1) for autophagic degradation [138].

TRIM21 promotes microglial polarization and astrogliosis in the CNS [44,56]. It also plays an important role in psoriasis, ubiquitinating and stabilizing keratin 17, which is a marker of keratinocyte hyperproliferation, and thus induces STAT3 activation [140]. At the same time, it enhances inflammation in keratinocytes of the psoriatic epidermis through the ubiquitination of the NF-κB p65 subunit [141]. Moreover, *TRIM21* knockout alleviates inflammation in the imiquimod-induced psoriasis-like skin inflammation mouse model [142]. However, TRIM21 negatively regulates inflammation in patients with inflammatory bowel diseases. Interferon regulatory factor 3 was recognized as its downstream target that mediates CD4+ T-cell differentiation into TH1 and TH17 cells, a process that is inhibited by the ectopic expression of *TRIM21* [139]. It was also shown that TRIM21 mediates the downregulation of active IKK beta and, consequently, NF-κB signaling in vitro [143].

TRIM22 is implicated in neuroinflammation and tumor progression by activating NF-κB signaling in response to stressors like OGD and TNF-α treatment [94,103]. The role of TRIM22 in rheumatoid arthritis is also pro-inflammatory, acting via the NF-κB pathway. The protein leads to excessive proliferation and inflammation in fibroblast-like synoviocytes, and is potentially activated via FOXC1 [145]. Moreover, TRIM22 enhances cell proliferation and inflammation in psoriasis through the PI3K/AKT/mTOR pathway [144]. In vitro experiments have proven the ability of TRIM22 to activate the NF-κB pathway in macrophage cell lines and HEK293T cells, but specifically downregulates TRAF6-induced NF-κB activation in HEK293T cells via the degradation of TAB2 [146].

TRIM27 exhibits ambiguous roles in neuroinflammation, being increased in hypoxic–ischemic encephalopathy, while its downregulation reduces brain infarct volume and inflammation [76], yet simultaneously acting neuroprotectively in ischemic stroke models [77]. Its role in other inflammatory processes is also diverse. For instance, according to the data from the literature, it plays a pro-inflammatory role in a mouse model of asthma through NLRP3 inflammasome activation, promotes sepsis-induced inflammation through the ubiquitination of PPARγ, and induces inflammation through the activation of STAT3 signaling [150,151,152]. However, in other cases it opposes inflammation. Thus, TRIM27 suppresses inflammation in pediatric pneumonia through downregulation of the TLR4/NF-κB signaling pathway, and in cardiac I/R injury by negatively regulating p53 [76,148].

TRIM28 exemplifies the role of TRIM proteins in microglial activation, enhancing microglial glycolysis and activation in sepsis [78], while also orchestrating ferroptosis, thereby promoting neuroinflammation and neuropathic pain [79]. It also promotes the nuclear accumulation of α-Syn and tau in murine neurons, thus exacerbating inflammation [47]. In MEF and HEK 293T cell lines, TRIM28 was proven to facilitate type 1 interferon activation by mediating the K63-linked ubiquitination of TBK [156]. In mouse primary peritoneal macrophages treated with LPS, TRIM28 SUMOylates NLRP3, and thereby activates inflammasomes [155]. In the context of necroptosis, S473-phosphorylated TRIM28 promotes the activation of inflammatory genes in human colorectal adenocarcinoma cell line HT-29 [154]. At the same time, TRIM28 acts as SUMO ligase for TRAF6 upon Hepatitis B virus infection, and the TRIM28-mediated SUMOylation of TRAF6 inhibits NF-κB activation and inflammation in a hydrodynamic injection Hepatitis B virus mouse model [153].

TRIM29 was proven to have anti-inflammatory effects in the CNS and pro-inflammatory effects outside its borders. Interestingly, in both cases, its functions are related to inflammasome regulation. In the murine OGD model of ischemic stroke, it decreases inflammation, K48-ubiquitinating NLRC4 and mediating its degradation [105]. At the same time, in murine intestinal epithelial cells subjected to poly I:C or enteric RNA viruses, TRIM29 was shown to promote inflammation by promoting the K-48 linked ubiquitination of NLRP6 and NLRP9b and their degradation, and to decrease the level of the anti-inflammatory cytokine IFN-λ [157]. In high-glucose-treated murine podocytes, TRIM29 is overexpressed and promotes pyroptosis via activating the NF-κB/NLRP3 pathway [158].

TRIM31 mediates the anti-inflammatory effects of parthenolide and MDZXYS by promoting the ubiquitination and degradation of the NLRP3 inflammasome in microglia [80,81]. Similarly, its role outside the CNS is also mainly protective due to its ability to suppress the NLRP3. *TRIM31* deficiencies have been reported as detrimental in alum-induced peritonitis, causing airway inflammation in asthma and also affecting dextran sulfate sodium-induced colitis [13,159,160]. At the same time, TRIM31 targets TIGAR for degradation, which increases ROS and inflammation in neurons under conditions of ischemic brain injury. TIGAR is predominantly expressed in neurons, which makes the effects of TRIM31 cell type-dependent [106].

TRIM32 plays a dual role in neuroinflammation and neuroprotection, since it supports axonal regeneration and decreases the production of inflammatory cytokines [82], suppresses microglia pyroptosis through the downregulation of NEK7 [83], and also increases BBB permeability in the context of *Streptococcus suis* meningitis [84]. Outside the CNS, the role of TRIM32 is predominantly pro-inflammatory. Thus, it aggravates the inflammation in rheumatoid arthritis, increasing the production of pro-inflammatory cytokines, activating the NF-κB signaling pathway and interacting with TRAF2 [161]. As in OGD/reoxygenation-treated hippocampal neurons, its knockdown attenuates inflammation in high-glucose-induced podocyte injury via the activation of NRF2 signaling and, consequently, the modulation of the AKT/GSK-3β axis [163]. It was also discovered to promote the inflammatory factor-induced apoptosis of rat nucleus pulposus cells via the ubiquitination of AXIN1, which triggers β -catenin signaling. It is also associated with the severity of intravertebral disc disease [190]. TRIM32 promotes the degradation of DPEP2 in macrophages and the subsequent activation of NF-κB and p38 MAPK signaling [164]. In psoriasis, TRIM32 binds to the PIASy protein, and therefore enhances the disease, increasing the levels of CCL20 and fueling the positive feedback loop of CCL20 and Th17 activation [165]. However, in patients with atopic dermatitis, which is a Th2-dominant disease, TRIM32 levels in the skin are low, and *TRIM32* knockout in mice skews the immune response towards Th2 activation, which results in atopic dermatitis-like phenotypes [162].

TRIM47 manifests itself as an inflammation-related factor not only in the CNS. TRIM47 aggravates LPS-induced acute lung injury via the activation of NF-κB and MAPK signaling pathways, as well as the K63-linked ubiquitination of TRAF2 pathway [11]. In human bronchial epithelial cells, TRIM47 was shown to enhance house dust mite-induced pyroptosis by the K63-linked ubiquitination of NEMO [12]. Similarly, TRIM47 exacerbates neuroinflammation via NF-κB signaling; however, it also exhibits a neuroprotective effect through NRF2 activation that is specific to the CNS [111].

TRIM56 mitigates PANoptosis after SCI by promoting the ubiquitination of YBX1, thereby regulating ZBP1 expression and alleviating neuroinflammation [112]. Outside the CNS, TRIM56 participates in various pathways related to inflammation. In intervertebral disc degeneration, disassembly of the complex of TRIM56 with ATR leads to the proteasome degradation of ATR and the subsequent activation of cGAS/STING axis-dependent inflammatory response, which makes TRIM56 an anti-inflammatory factor resisting nucleus pulposus degeneration [174]. At the same time, TRIM56 positively regulates TNF-α-induced NF-κB signaling via the ubiquitination of TAK1 in HEK293T and A549 cells [175].

TRIM59 acts anti-inflammatorily, mediating the ubiquitination of NLRP3 in microglia [88]. It downregulates complement C3 expression by macrophages, which supposedly affects the changes in clusterin expression [176]. TRIM59 also acts anti-inflammatorily in osteoarthritis, attenuating IL-1β-driven cartilage matrix degradation through the suppression of JAK2/STAT3 signaling pathways [177]. However, in lung cancer, TRIM59, on the other hand, promotes inflammation and activates macrophages via the activation of the NLRP3 inflammasome [178].

TRIM67 exhibits anti-inflammatory properties in the CNS by modulating ubiquitination pathways and inhibiting NF-κB signaling, thereby reducing inflammation in various neurological conditions [55,115]. In HEK293T and mouse embryonic fibroblasts, TRIM67 is shown to interrupt β-TrCP-mediated IκBα degradation by competing with IκBα for β-TrCP binding, which results in the inhibition of NF-κB signaling [181]. In mice with obesity, when fed a high-fat diet, TRIM67 ameliorates intestinal inflammation [182] as well as hypothalamic neuroinflammation [115]. Despite abundant evidence supporting the anti-inflammatory properties of the protein, there is evidence to the contrary; TRIM67 activates hepatic inflammation induced by obesity [183].

TRIM72 exhibits neuroprotective and anti-inflammatory effects by blocking LPS-induced neuroinflammation through TLR4/NF-κB and NLRP3/Caspase-1/IL-1β pathways, while also alleviating neuropathic pain and oxidative stress via NRF2/HO-1 signaling, and it can traverse the BBB during neuroinflammation [116,117,118,119]. In striated muscles, the main functions of TRIM72 revolve around the sarcolemmal repair process. This is why autoantibodies targeting TRIM72 impair the membrane barrier function, which leads to the release of autoantigens and the further progression of inflammatory myopathy [184]. TRIM72 is also able to resolve muscle inflammation via the deactivation of the NLRP3 inflammasome [185]. It was discovered that TRIM72 repairs the cell membrane not only in muscle cells, but also in alveolar epithelial cells, and thus ameliorates lung fibrosis [187]. However, the role of TRIM72 is not always protective. Thus, in a *P. aeruginosa*-induced murine model of pneumonia, *TRIM72* overexpression promotes bacteria-induced NF-κB activation in alveolar macrophage cells, while the genetic ablation of *TRIM72* decreases inflammation and increases the survival of the animals. This effect is caused by the ability of TRIM72 to inhibit the complement receptor of the Ig superfamily in alveolar macrophages [186].

Such contradictory findings regarding the roles of the aforementioned TRIM proteins highlight the necessity of additional research.

### 7.5. TRIMs with Insufficient Data

TRIM69 protects against inflammation and apoptosis in high-fat-diet-treated mice and LPS-treated microglia by deactivating ASK-1 [91]. Unfortunately, there are not many data covering the role of TRIM69 in inflammatory processes outside the CNS. However, there is evidence of its increased expression in rectum biopsy samples derived from patients with inflammatory bowel disease [122].

As the TRIM family includes numerous members, other TRIM proteins not discussed in this review might also participate in inflammation; however, no relevant studies have been reported to date. Therefore, all remaining members of the TRIM family can currently be classified as TRIMs with insufficient data.

While the specific pathways and cell types involved differ between neuroinflammation and general inflammation, the overarching theme is the same, namely, TRIM proteins are crucial regulators of the immune response. A vast majority of TRIMs that are involved in the regulation of CNS inflammation are also involved in other inflammation-related pathologies. A comparison of the mechanisms of action of TRIM proteins shows that even when a TRIM protein exhibits the same properties (pro- or anti-inflammatory) in brain and other tissues by acting on the same signaling pathway, the specific mechanisms may differ, suggesting unique evolutions depending on the cellular context. This statement is supported by the existence of many TRIMs with divergent properties. Our conclusion is in line with the review by Luiting Yang and Haibin Xia on the multifaceted role of TRIM proteins in orchestrating inflammation. The authors have emphasized the context-dependent manner of TRIM regulation, which is affected by such factors as species, cell context and microenvironment [6]. Keeping that in mind, a cautious approach should be adopted in using TRIM family members as prospective targets in drug design, since even the same protein may impact inflammation in different ways under different circumstances.

In the present review, we have strived to examine the currently available, published data on the involvement of TRIM proteins in neuroinflammation, taking into account the type of inflammation and brain cell types. We also attempted to explore correlations with data on inflammation outside of the CNS, in order to reveal patterns and highlight areas where further research is needed.

## 8. TRIM Proteins and Systemic Inflammatory Control

The innate recognition of pathogens or damage drives the production of pro-inflammatory mediators and the up-regulation of co-stimulatory molecules on antigen-presenting cells [191]. The available data indicate that TRIM proteins act as important regulators linking inflammation in situ to systemic and adaptive immune responses by several mechanisms [192,193]. TRIM27, for example, associates with PI3KC2β and promotes its K48-linked ubiquitination and degradation, which downregulates PI3K signaling and T-cell activation [194]. TRIM28 is required for normal IL-2 production and for maintaining physiological numbers of CD4^+^ and CD8^+^ T cells; mice lacking TRIM28 accumulate autoreactive Th17 cells and develop spontaneous inflammatory disease [195]. TRIM24 is preferentially expressed in Th2 cells, and its absence leads to the reduced production of IL-5, IL-13 and IL-10 together with defective IL-1R expression and signaling, resulting in weaker Th2 activation and attenuated type-2 allergic inflammation [196]. TRIM32 promotes the expression of the chemokine CCL20 in keratinocytes, and supports Th17-driven, psoriasis-like skin inflammation [162]. Trim21-null mice develop dermatitis and systemic autoimmunity characterized by increased IL-17, IL-6 and IL-23 production and dependence on the IL-23–Th17 axis [197]. Recent work further implicates TRIM21 in immune-checkpoint regulation. The protein catalyzes the K63-linked ubiquitination of PD-1 at lysine 233, counteracting its K48-linked ubiquitination and proteasomal degradation, and thus stabilizing PD-1 on T cells [198]. This coinhibitory receptor inhibits T cells and limits IL-2 production and the infiltration of immune cells, thus preventing autoimmune tissue damage [199].

Our understanding of how these mechanisms operate in neuroinflammatory and neurodegenerative diseases is still incomplete. Nevertheless, we analyzed the existing literature on those TRIM proteins that, in our classification, were assigned to groups with a unidirectional (pro- or anti-inflammatory) effect on inflammation in the CNS and peripheral tissues. We then assessed whether they also exert a similarly unidirectional influence on the systemic inflammatory response, excluding the data concerning oncological diseases.

The silencing of TRIM8 inhibited Th2 development and Th2 cytokine secretion in ovalbumin (OVA)-treated mice as a model of allergic asthma [129]. Mutations in TRIM37 lead to Mulibrey (muscle–liver–brain–eye) syndrome, an autosomal recessive disorder. The authors observed functional defects (impaired activation and cytokine production) in CD4+ T cells in MUL (only one patient), and assumed a role of TRIM37 in the immune response of T lymphocytes [200]. This is consistent with TRIM8 and TRIM37’s pro-inflammatory roles.

TRIM 9 plays a protective, immunoregulatory role in myasthenia gravis, an organ-specific autoimmune neuromuscular disorder [201]. TRIM11 causes AIM2 ubiquitination and degradation in CD4+ T cells, thereby suppressing the stability of Treg cells during EAE [202]. At the same time, evidence exists that AIM2 is located in the nucleus of Th17 cells, where it interacts with RORγt, enhancing its binding to the Il17a promoter. A recent study has revealed that AIM2 promotes Th17 cell differentiation and IL-17A production [203], and enhances the production of other proinflammatory mediators (IL-6, IL-1β, TNF-α, and chemokines). The results are in line with TRIM9 and TRIM11’s protective and anti-inflammatory roles.

There is no direct information on the regulation of adaptive immunity by TRIM3, TRIM6, TRIM14, TRIM52, TRIM62, and TRIM65. The impacts of these proteins on systemic inflammation are mediated through the innate immune response and cytokine production. Taken together, none of the TRIM proteins that, in our classification, were assigned to groups with a unidirectional (pro- or anti-inflammatory) effect on inflammation in the CNS and peripheral tissues meet the exclusion criteria.

## 9. Conclusions and Future Directions

TRIM proteins are important modulators of inflammation, forming a regulatory network with both pro- and anti-inflammatory properties.

For several TRIM proteins, intracellular localization has been identified and shown to correlate with their function, as it defines the spatial accessibility of interacting molecules involved in the regulation of inflammation.

Among the major mechanisms underlying the pro- and anti-inflammatory roles of TRIM proteins in both the CNS and peripheral tissues, the NF-κB and NLRP3 inflammasome signaling pathways predominate. The involvement of TRIMs as repressors of transcription has also been described. The modulation of inflammation via effects on misfolded proteins appears to be specific to neurodegeneration. By contrast, the influences of TRIM proteins on cGAS/STING signaling have been studied predominantly in non-neuronal cells during viral infection; however, emerging evidence suggests that this pathway also contributes to neuroinflammation.

Within the CNS, TRIMs exhibit functional diversity across all major cell types; microglia, neurons, and astrocytes. The predominance of data regarding TRIM’s function in microglia is reasonable, given that these cells serve as the key immune cells of brain tissue. There is also evidence of TRIM’s effects on neurons, primarily in relation to neuronal survival and oxidative stress, rather than the direct regulation of intracellular inflammatory signaling. Despite the fact that astrocytes are recognized as immunocompetent cells, the information on TRIM’s actions in these cells is currently limited.

The available data from the literature confirm that many TRIM proteins exert similar effects, either pro-inflammatory (TRIM6, TRIM8, TRIM14, TRIM37, TRIM52, TRIM62) or anti-inflammatory (TRIM3, TRIM9, TRIM11, TRIM65), in neuroinflammation as well as in the inflammatory pathology in the body, although, in some cases, different specific mechanisms of action have been described. For certain TRIMs, the published evidence regarding their role in neuroinflammation versus inflammation in other tissues reflects the opposite (TRIM24, TRIM44, TRIM45) or something contradictory (TRIM13, TRIM20, TRIM21, TRIM22, TRIM27, TRIM28, TRIM29, TRIM31, TRIM32, TRIM47, TRIM56, TRIM59, TRIM67, TRIM72). For TRIM69, there are insufficient data for such a comparison, indicating that further studies are needed. A number of other TRIMs are not mentioned in the published data in the context of inflammation. The knowledge of TRIM proteins’ involvement in adaptive immunity and systemic inflammation is expanding, but remains limited.

Targeting pro-inflammatory or boosting anti-inflammatory TRIMs may have therapeutic effects in the context of neuroinflammation. Recent studies illustrate their potential. TRIM9 gene therapy restores NF-κB balance in the context of brain injuries, such as ischemic stroke [54]. A protective and regenerative role for recombinant human TRIM72 [36] and TRIM28 inhibitors is suggested in the context of neurodegeneration [47]. In a mouse model of PD, recombinant TRIM11 injection significantly reduced α-Syn pathology [204].

Neurodegenerative diseases are frequently accompanied by comorbidities whose pathogenesis also involves inflammation and often share common dysregulated inflammatory pathways with neurodegeneration [3]. Because TRIM proteins are implicated in a wide range of immune and other peripheral processes, in addition to their roles in the CNS [8], the systemic administration of TRIM-targeted compounds is expected to influence peripheral TRIM-dependent pathways, which in turn might indirectly modify brain homeostasis through immune and cytokine signaling. We propose that, when selecting a TRIM protein as a target for the modulation of neuroinflammation, it might be important to take into account the available data on its effects on the inflammation in other organs and tissues, in order to avoid possible undesirable systemic effects and derive additional therapeutic benefits. Alternatively, intracerebroventricular injections could possibly be used instead of systemic administration [205].

It is important to emphasize other potential challenges of therapeutic applications. The central role of TRIMs in multiple signaling cascades involved in the inflammatory response [193] increases the risk of undesirable adverse effects. A number of TRIMs function as E3 ubiquitin ligases within the ubiquitin–proteasome system. Altering one TRIM can reshape other E3s’ activity and consequently change the key inflammatory pathways, such as NF-κB or others, which complicates the prediction of specific effects. TRIM-targeted therapeutics for neuroinflammation ideally should be brain-specific and, where possible, cell type- or isoform-selective. A thorough preclinical evaluation is required to detect subtle off-target effects.

A specific difficulty that is to be addressed is the delivery of TRIM-related compounds to the CNS. One of the possible solutions is to use specific virus vectors that are able to cross the BBB and have a tropism to CNS tissues for gene therapy [206]. This strategy was successfully applied in vivo with TRIM9 and TRIM11 gene therapies [54,204]. Neuron-specific AAV vectors have been used in vivo to selectively overexpress Trim32 in spinal cord neurons, demonstrating proof-of-concept for cell type-specific targeting [207]. The silencing of TRIMs in therapy could be achieved via siRNA, which, however, raises its own challenges related to the design of delivery systems [208]. Nanoparticle-based delivery methods are another rapidly developing strategy, which has shown promise in the context of drug delivery to the CNS [209].

Another important challenge is the limited understanding of several TRIM proteins in the CNS. The lack of data regarding astrocytes is particularly evident. Addressing these gaps might be essential to developing a thorough understanding of TRIMs and their potential therapeutic implications in brain disorders. In this review, we have attempted to identify key gaps and opportunities for future studies.

## Figures and Tables

**Table 1:** Published data directly linking a specific intracellular TRIM protein’s localization to its specific role in inflammation.

Cellular Compartment	Examples	
Cytoplasm	TRIM9	Is associated with intracellular cytoskeletons, then upon binding to β-TrCP, is redistributed into cytoplasm [17,40]
	TRIM14	Inhibits cGAS degradation facilitating antiviral innate immune response [41]
	TRIM21	Acts as a negative regulator of the cytosolic DNA sensor DDX41 [26]
	TRIM38	Interacts with TAB2 and promotes the translocation of TAB2 to the endosomes/lysosomes for proteolysis; prevents cGAS polyubiquitination and degradation [27,42]
	TRIM45	Is localized as diffuse dots in the cytoplasm, upregulates the activity of NF-κB, ubiquitinates TAB2, activating TAK1-NF-κB [10,30]
	TRIM47	Ubiquitinates TRAF2, activating NF-κB and MAPK signaling pathways [11]
	TRIM56	Is a crucial component of the cytosolic DNA sensing pathway that induces anti-DNA viral innate immunity; forms a complex with TRIF to promote the downstream activation of IRF3 and NF-κB [31,43]
Nucleus	TRIM14	Acts as an epigenetic regulator through suppressing histone H3K9 trimethylation by preventing the histone demethylase KDM4D from degradation, which leads to the expression of the KDM4D-directed pro-inflammatory cytokines [33]
	TRIM21	Transports PKM2 into the nucleus, where PKM2 phosphorylates STAT3 and NF-κB, enhancing the pro-inflammatory response [44]
	TRIM24	Acts as a repressor of repetitive DNA elements, TRIM24-repressed targets include several genes involved in inflammatory response (*COX2, TNFAIP3*) [34]
	TRIM28	The inhibition of SETDB1–TRIM28 results in the formation of micronuclei in the cytoplasm, which is known to activate the cGAS–STING pathway [45]
	TRIM41	Full-length TRIM41alpha and TRIM41beta are both observed as speckles in the cytoplasm and the nucleus; regulates neuroinflammation, decreasing *SNCA* transcription via transcription factor ZSCAN21 [32,46]
Mitochondria	TRIM14	Localizes to the outer mitochondrial membrane, interacting with MAVS and NEMO [35]
Membranes	TRIM38	Negatively regulates TNF-α- and IL-1β-induced signaling by mediating the lysosome-dependent degradation of TAB2/3, translocates TAB2 to the lysosome [27]
	TRIM72	Recombinant TRIM72 protects various cell types against membrane disruption, including neuronal and immune origin cells [36,37]
Dynamic Localization	TRIM8	Resides in the nucleus at rest, but upon TNF-α stimulation, translocates to the cytoplasm, where it ubiquitinates TAK1 and enhances NF-κB signaling [39]
	TRIM28	Promotes the nuclear accumulation of α-synuclein and tau; may regulate both its own and its substrates’ subcellular localization through post-translational modifications [47]
	TRIM32	Under PD stress conditions, migrates from cytoplasm to mitochondria, where it degrades MYC and promotes the apoptosis of dopaminergic neurons in PD [38]
	TRIM38	Is found in the membrane fraction in unstimulated cells, and TNF-α or IL-1β stimulation promotes the translocation of TRIM38 from the cytosol to the membrane fraction. TRIM38 interacts with TAB2 in both the cytosol and the endosome membrane fractions, and promotes the translocation of TAB2 to the endosomes/lysosomes for proteolysis [27]

**Table 3:** Comparison of published data on TRIM proteins’ functions in neuroinflammation versus inflammation in other organs and tissues.

TRIM Protein	Role in CNS			Role Outside CNS			
	Model Cell/Tissue Type	Effect on Inflammation		Model Cell/Tissue Type	Effect on Inflammation		Functional Concordance
TRIM3	Mice, PD model; SH-SY5Y cells	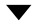	[97]	Rats, LPS-induced acute kidney injury	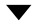	[123]	Anti-inflammatory
TRIM6	Mice, PD model; neurons	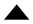	[98]	Mice, influenza A virus infection, lung epithelium	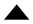	[124]	Pro-inflammatory
	PD patients and their healthy twins/siblings, peripheral blood mononuclear cells	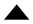	[125]	Human kidney-2 (HK2) cells	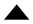	[126]	
TRIM8	Mice, OGD model, microglia	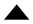	[73]	Osteoarthritis, human chondrocytes	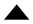	[127]	Pro-inflammatory
	Mice, IR injury, astrocytes	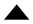	[72]	Mice, *Pseudomonas aeruginosa*-induced keratitis, cornea	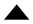	[9]	
				Mice, dextran sulfate sodium-induced colitis, colonic tissue	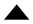	[128]	
				Mice, allergic asthma	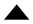	[129]	
TRIM9	Rats, streptozocin-induced model of AD	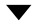	[99]	Human HEK293T, A549, TNF-α or IL-1β-stimulated	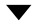	[17]	Anti-inflammatory
	Neuroblastoma SK-N-AS cells, TNF-α or IL-1β-stimulated	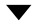	[17]				
	Mice, MCAO, neurons	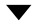	[54]				
TRIM11	AD patients, postmortem brain tissues	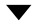	[100]	TRIM11-overexpressing THP-1 macrophages	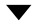	[130]	Anti-inflammatory
TRIM13	Mice, high-fat-diet-induced neuroinflammation, cortex, hippocampus and hypothalamus tissues	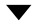	[101]	TNF-α stimulated HEK293, HeLa, A549 and MCF7 cell lines	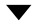	[131]	Divergent evidence
				HeLa cell line transfected with Nur77 and Trim13	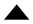	[132]	
				HEK293T cells transfected with SARS-CoV-2 protein	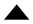	[133]	
TRIM14	Rats, model of cerebral I/R, hippocampal tissue	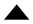	[59]	HSV-1-exposed THP-1 cells;	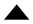	[41]	Pro-inflammatory
	Mice, EAE, brain and spinal cord	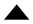	[33]	Human vascular endothelial cells, LPS or TNF-α stimulation	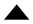	[134]	
				HeLa, human BMDMs, LPS or TNF-α stimulation	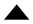	[135,136]	
TRIM20	Mice *becn1-KO*, TBI, cortex	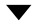	[74]	Human neutrophils, monosodium urate-stimulated	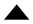	[137]	Divergent evidence
				THP-1 cells; human peripheral blood monocyte-derived macrophages, LPS stimulation	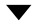	[138]	
TRIM21	Human cell line SHSY-5Y in the presence of picomolar concentrations of tau seeds	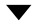	[102]	Patients with inflammatory bowel diseases, CD4+ T cells from peripheral blood	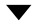	[139]	Divergent evidence
	Mice, autoimmune encephalomyelitis, astrocytes	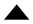	[44]	Psoriasis, primary human keratinocytes, human keratinocyte cell line HaCaT cells	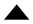	[140,141]	
	Human microglial cells (CHME3), Japanese encephalitis virus (JEV) infection	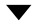	[75]	Mice, imiquimod-induced psoriasis-like skin inflammation model	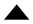	[142]	
				HEK cell line transfected with the NF-κB reporter plasmid	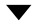	[143]	
TRIM22	Mice, ischemic cortex tissues OGD/R treated HCN-2 neurons	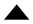	[103]	Psoriasis, macrophage cell lines and HEK293T cells	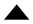	[144]	Divergent evidence, mostly pro-inflammatory
				Rheumatoid arthritis, fibroblast-like synoviocytes	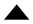	[145]	
				HEK293T cell line transfected with TRIM22 and TRAF6	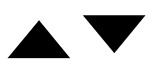	[146]	
TRIM24	Triple transgenic AD (3xTg-AD) mice treated with esketamine	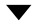	[104]	Mice, LPS-induced endotoxic shock, macrophages	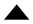	[147]	Opposite effects
TRIM27	Mice, MCAO/R; microglial cells exposed to OGD/R	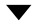	[77]	Mice, myocardial I/R, primary cardiomyocytes	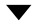	[148]	Divergent evidence
	Microglia, hypoxic-ischemic encephalopathy model	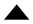	[76]	LPS-treated human WI-38 lung fibroblast cell line, a model of pediatric pneumonia	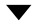	[149]	
				LPS-induced septic mice, lung tissues	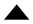	[150]	
				Human keratinocyte HaCaT cells, treated with IL-6	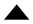	[151]	
				Mice, model of asthma; MLE12 cells *KO-TRIM27*	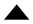	[152]	
TRIM28	Mice, sepsis model, BV2 microglial cells transfected with TRIM28	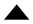	[78]	Mice, hydrodynamic injection HBV model	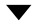	[153]	Divergent evidence, mostly pro-inflammatory
	SH-SY5Y cells transfected with α-Syn and tau	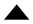	[47]	TNFα-stimulated FADD-deficient Jurkat cells	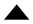	[154]	
	Mice, NPP model, microglia	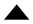	[79]	Mice, LPS-treated primary peritoneal macrophages	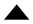	[155]	
				MEF and HEK 293T cell lines co-transfected with TRIM28 and TBK1 subjected to polyI:C	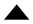	[156]	
TRIM29	Mice, OGD model of ischemic stroke	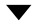	[105]	Murine intestinal epithelial cells subjected to polyI:C or enteric RNA viruses	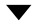	[157]	Divergent evidence
				Mice, glucose-treated podocytes	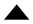	[158]	
TRIM31	Mice, CUMS depression model, prefrontal cortex, hippocampus	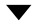	[81]	Alum-induced peritonitis, airway inflammation in asthma, and dextran sodium sulfate-induced colitis	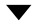	[13,159,160]	Divergent evidence, mostly anti-inflammatory
	LPS/IFN-γ-induced BV2 and HMC3 microglia	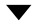	[80]				
	Rat, PC12 cells stimulated by OGD/R	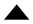	[106]				
TRIM32	Mice, spinal cord injury, astrocytes and microglia	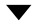	[82]	Rheumatoid arthritis, TNF-α treated fibroblast-like synoviocytes	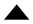	[161]	Divergent evidence
	LPS-induced murine microglial cell line BV-2	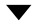	[83]	Mice, atopic dermatitis model	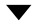	[162]	
	Mice, meningitis caused by *Streptococcus suis*	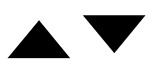	[84]	Mice, high glucose (HG)-induced injury, podocytes	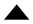	[163]	
	OGD/R, primary mouse hippocampal neurons	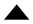	[38]	LPS-activated macrophages iBMDMs	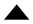	[164]	
	SH-SY5Y dopaminergic neuronal cell line, PD stress conditions (rotenone and 6-OHDA)	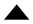	[107]	Human keratinocyte line HEKnV treated with Th17 (IL-17A, TNFα) cytokines	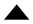	[165]	
TRIM37	Mouse microglial BV-2 cell line; MCAO-induced rat brain damage	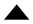	[69,85]	Hepatic I/R injury	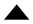	[166]	Pro-inflammatory
				LPS-induced A549 cell injury model	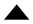	[167]	
				Viral infection in vivo	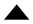	[168]	
TRIM44	Rats, TBI	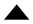	[108]	Mice, renal I/R injury Renal cells subjected to hypoxia/reoxygenation	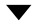	[169]	Opposite effects
TRIM45	Primary microglia with OGD/R treatment	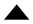	[10]	HEK293 cell line stimulated with TNF-α	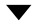	[30]	Opposite effects
	BV2 cells treated with LPS/ATP	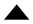	[86]				
TRIM47	Human primary brain microvascular endothelial cells; Mice, *TRIM47*-deficient	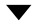	[111]	HUVECs, TNF-α stimulated	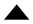	[11]	Divergent evidence, mostly pro-inflammatory
	Rat neuronal cell line H19-7, sevoflurane-treated	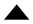	[110]	Human bronchial epithelial cell line BEAS-2B; house dust mite-induced pyroptosis	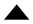	[12]	
	Rats, I/R, brain samples, SH-SY5Y cells OGD-treated	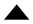	[109]				
TRIM52	Rats, microglia from cerebral cortex activated by LPS	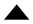	[87]	Human periodontal ligament cells stimulated by LPS	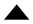	[170]	Pro-inflammatory
				Mice, sulfate sodium-induced inflammatory bowel disease, colon tissue	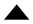	[171]	
				IL-1β-treated synovial fibroblasts of patients with osteoarthritis	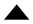	[172]	
				HEK293T cell line treated with TNF-α	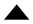	[173]	
TRIM56	Mice, SCI, primary neurons	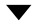	[112]	Humans, spinal pathology, nucleus pulposus cells	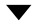	[174]	Divergent evidence
				HEK293T and A549 cells stimulates by TNF-α	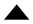	[175]	
TRIM59	Human microglial HMC3 cells, OGD/R	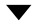	[88]	Mouse bone marrow-derived macrophages	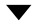	[176]	Divergent evidence, mostly anti-inflammatory
				Primary human OA (osteoarthritis) chondrocyte culture, IL-1β treated	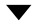	[177]	
				THP-1 macrophages stimulated with LPS/ATP	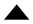	[178]	
TRIM62	Mice, I/R, brain samples; Mice, microglial BV-2 cell line, OGD; Mice, high-fat-diet/streptozotocin-induced diabetes, hippocampus tissues	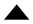	[89,90]	Mice, dextran sulfate sodium model of intestinal inflammation	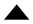	[179]	Pro-inflammatory
				Patients with ICU-acquired weakness, myocytes treated with LPS	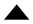	[180]	
				HEK293 cell line treated with TNF-α and LPS	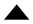	[22]	
TRIM65	Mice, CUMS model of depression, hippocampus tissues hippocampus tissues	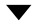	[114]	THP-1 cell line, murine BMDMs stimulated with NLRP3 agonists	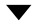	[15]	Anti-inflammatory
	Mice, MCAO model of stroke	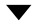	[113]				
TRIM67	Primary cultured microglia, MCAO/R model	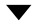	[55]	HEK293T cell line, primary mouse embryonic fibroblasts	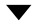	[181]	Divergent evidence, mostly anti-inflammatory
	Murine hypothalamus; obesity	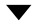	[115]	Porcine intestinal cells (IPEC-J2) stimulated with palmitic acid	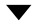	[182]	
				Mice with obesity, high-fat-diet-induced non-alcoholic fatty liver disease	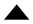	[183]	
TRIM69	Mice, high-fat diet model; Mouse microglial BV-2 cell line, LPS-treated	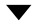	[91]	NA	NA		Insufficient data
TRIM72	LPS-treated HT22 cell line	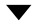	[116]	Mice, FDB skeletal muscles exposed to laser injury	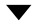	[184]	Divergent evidence, mostly anti-inflammatory
	Rats, cerebral I/R injury, brain tissue	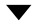	[119]				
				AAV-TRIM72 mdx mice	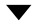	[185]	
	Mice, LPS-treated, hippocampus	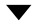	[117]	Mice, *P. aeruginosa* -induced model of pneumonia, alveolar macrophage cells	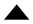	[186]	
	Rats, chronic constriction injury, spinal cord	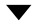	[118]	Idiopathic pulmonary fibrosis (IPF), immortalized and primary alveolar epithelial ATII cells	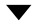	[187]	
	Mouse brain tissue and primary rat cortex neurons treated with Aβ42 or cerebrospinal fluid from AD patients	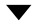	[36]				

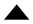
 symbols indicate pro-inflammatory effects, 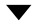 symbols indicate anti-inflammatory effects.

## Data Availability

No new data were created or analyzed in this study. Data sharing is not applicable to this article.

## Abbreviations

The following abbreviations are used in this manuscript:

AD	Alzheimer’s disease
AP-1	activating protein-1
BBB	blood–brain barrier
CC	Coiled-Coil domain
CNS	central nervous system
DAMPs	danger-associated molecular patterns
EAE	experimental autoimmune encephalomyelitis
IKK	IκB kinase
I/R	ischemia-reperfusion
LPS	lipopolysaccharide
MCAO/R	middle cerebral artery occlusion/reperfusion
NEMO	NF-κB essential modulator
NF-κB	nuclear factor-kappa B
NLRP	NOD-like receptor family pyrin domain-containing inflammasome
NSP6	nonstructural protein 6
OGD/R	oxygen-glucose deprivation/reperfusion
PAMPs	pathogen-associated molecular patterns
PD	Parkinson’s disease
ROS	reactive oxygen species
SCI	spinal cord injury
SUMO	Small Ubiquitin-like Modifier
α-Syn	α-synuclein
TBI	traumatic brain injury
TRAF2	TNF receptor-associated factor 2
β-TrCP	β-transducin repeat-containing protein
TRIM	Tripartite motif
